# Supplementary material for: Biomarkers Correlated with Tuberculosis Preventive Treatment Response: A Systematic Review and Meta-Analysis
Source: Microorganisms. 2023 Mar 14;11(3):743. doi: 10.3390/microorganisms11030743 (PMC10057454; doi:10.3390/microorganisms11030743)
Supplement: Supplementary file 1 [file microorganisms-11-00743-s001.zip › microorganisms-2247626-supplementary.pdf]

Supplementary Table S1 PRISMA 2020 item checklist.

Section and Topic	Item #	Checklist item	Location where item is reported
**TITLE**			
Title	1	Identify the report as a systematic review.	Page 1
**ABSTRACT**			
Abstract	2	See the PRISMA 2020 for Abstracts checklist.	Page 1
**INTRODUCTION**			
Rationale	3	Describe the rationale for the review in the context of existing knowledge.	Pages 1-2
Objectives	4	Provide an explicit statement of the objective(s) or question(s) the review addresses.	Page 2
**METHODS**			
Eligibility criteria	5	Specify the inclusion and exclusion criteria for the review and how studies were grouped for the syntheses.	Page 3
Information sources	6	Specify all databases, registers, websites, organisations, reference lists and other sources searched or consulted to identify studies. Specify the date when each source was last searched or consulted.	Page 2
Search strategy	7	Present the full search strategies for all databases, registers and websites, including any filters and limits used.	Pages 2-3
Selection process	8	Specify the methods used to decide whether a study met the inclusion criteria of the review, including how many reviewers screened each record and each report retrieved, whether they worked independently, and if applicable, details of automation tools used in the process.	Page 3
Data collection process	9	Specify the methods used to collect data from reports, including how many reviewers collected data from each report, whether they worked independently, any processes for obtaining or confirming data from study investigators, and if applicable, details of automation tools used in the process.	Page 3
Data items	10a	List and define all outcomes for which data were sought. Specify whether all results that were compatible with each outcome domain in each study were sought (e.g. for all measures, time points, analyses), and if not, the methods used to decide which results to collect.	Page 3
	10b	List and define all other variables for which data were sought (e.g. participant and intervention characteristics, funding sources). Describe any assumptions made about any missing or unclear information.	Page 3
Study risk of bias assessment	11	Specify the methods used to assess risk of bias in the included studies, including details of the tool(s) used, how many reviewers assessed each study and whether they worked independently, and if applicable, details of automation tools used in the process.	Pages 3-4
Effect measures	12	Specify for each outcome the effect measure(s) (e.g. risk ratio, mean difference) used in the synthesis or presentation of results.	Page 4
Synthesis methods	13a	Describe the processes used to decide which studies were eligible for each synthesis (e.g. tabulating the study intervention characteristics and comparing against the planned groups for each synthesis (item #5)).	Page 4
	13b	Describe any methods required to prepare the data for presentation or synthesis, such as handling of missing summary statistics, or data conversions.	Page 4
	13c	Describe any methods used to tabulate or visually display results of individual studies and syntheses.	Page 4
	13d	Describe any methods used to synthesize results and provide a rationale for the choice(s). If meta-analysis was performed, describe the model(s), method(s) to identify the presence and	Page 4-5

Section and Topic	Item #	Checklist item	Location where item is reported
		extent of statistical heterogeneity, and software package(s) used.	
	13e	Describe any methods used to explore possible causes of heterogeneity among study results (e.g. subgroup analysis, meta-regression).	Page 4
	13f	Describe any sensitivity analyses conducted to assess robustness of the synthesized results.	None
Reporting bias assessment	14	Describe any methods used to assess risk of bias due to missing results in a synthesis (arising from reporting biases).	Page 4
Certainty assessment	15	Describe any methods used to assess certainty (or confidence) in the body of evidence for an outcome.	Pages 4
**RESULTS**			
Study selection	16a	Describe the results of the search and selection process, from the number of records identified in the search to the number of studies included in the review, ideally using a flow diagram.	Page 5
	16b	Cite studies that might appear to meet the inclusion criteria, but which were excluded, and explain why they were excluded.	Page 5
Study characteristics	17	Cite each included study and present its characteristics.	Page 5 and Table 1
Risk of bias in studies	18	Present assessments of risk of bias for each included study.	Page 5, Supplementary Table S3 and Figure S3
Results of individual studies	19	For all outcomes, present, for each study: (a) summary statistics for each group (where appropriate) and (b) an effect estimate and its precision (e.g. confidence/credible interval), ideally using structured tables or plots.	Page 6
Results of syntheses	20a	For each synthesis, briefly summarise the characteristics and risk of bias among contributing studies.	Page 6
	20b	Present results of all statistical syntheses conducted. If meta-analysis was done, present for each the summary estimate and its precision (e.g. confidence/credible interval) and measures of statistical heterogeneity. If comparing groups, describe the direction of the effect.	Page 6
	20c	Present results of all investigations of possible causes of heterogeneity among study results.	Page 6
	20d	Present results of all sensitivity analyses conducted to assess the robustness of the synthesized results.	None
Reporting biases	21	Present assessments of risk of bias due to missing results (arising from reporting biases) for each synthesis assessed.	Page 6
Certainty of evidence	22	Present assessments of certainty (or confidence) in the body of evidence for each outcome assessed.	Page 6
**DISCUSSION**			
Discussion	23a	Provide a general interpretation of the results in the context of other evidence.	Pages 6-7
	23b	Discuss any limitations of the evidence included in the review.	Page 8
	23c	Discuss any limitations of the review processes used.	Page 8
	23d	Discuss implications of the results for practice, policy, and future research.	Page 8
**OTHER INFORMATION**			
Registration and protocol	24a	Provide registration information for the review, including register name and registration number, or state that the review was not registered.	Page 2
	24b	Indicate where the review protocol can be accessed, or state that a protocol was not prepared.	None
	24c	Describe and explain any amendments to information provided at registration or in the protocol.	None
Support	25	Describe sources of financial or non-financial support for the	Page 8

Section and Topic	Item #	Checklist item	Location where item is reported
		review, and the role of the funders or sponsors in the review.	
Competing interests	26	Declare any competing interests of review authors.	Page 8
Availability of data, code and other materials	27	Report which of the following are publicly available and where they can be found: template data collection forms; data extracted from included studies; data used for all analyses; analytic code; any other materials used in the review.	Page 8

Supplementary Table S2 The search strategy for PubMed.

Number	Terms
1	tuberculosis infection
2	LTBI
3	latent tuberculosis infection
4	"Latent Tuberculosis"[Mesh]
5	latent TB
6	1 OR 2 OR 3 OR 4 OR 5
7	biomarkers [MeSH Terms]
8	biomarker
9	markers
10	marker
11	7 OR 8 OR 9 OR 10
12	prophylaxis
13	preventive therapy
14	prevention and control
15	prevention
16	preventive measures
17	preventive treatment
18	prophylactic treatment
19	prophylactic therapy
20	12 OR 13 OR 14 OR 15 OR 16 OR 17 OR 18 OR 19
21	6 AND 11 AND 20

((((((tuberculosis infection)) OR (LTBI)) OR (latent tuberculosis infection)) OR ("Latent Tuberculosis"[Mesh]) OR latent TB) AND ((((biomarkers [MeSH Terms]) OR (biomarker)) OR (markers)) OR (marker))) AND ((((((((prophylaxis) OR (preventive therapy)) OR (prevention and control)) OR (prevention)) OR (preventive measures)) OR (preventive treatment)) OR (prophylactic treatment)) OR (prophylactic therapy))

Supplementary Table S3 Summary of the QUADAS-2 risk of bias assessment.

Domain	Items	Study-specific										
		**Ying Du (2022)**	**Ock-Hwa Kim (2020)**	**Katie Ewer (2006)**	**Irene Andia Biraro (2015)**	**Elisa Petruccioli (2018)**	**Delia Goletti (2007)**	**Haoran Zhang (2020)**	**Xuefang Cao (2021)**	**SW Lee (2012)**	**Henan Xin (2020)**	**Mulugeta Belay (2021)**
Patient selection	Was a consecutive or random sample of patients enrolled?	Yes	Yes	No	Yes	No	Yes	Yes	Yes	No	Yes	No
	Was a case-control design avoided?	Yes	No	No	Yes	No	No	Yes	Yes	Yes	Yes	Yes
	Did the study avoid inappropriate exclusions?	Unclear	No	No	Yes	Yes	No	Yes	Yes	Yes	Yes	Yes
	Could the selection of patients have introduced bias?	Low	High	High	Low	High	High	Low	Low	Intermediate	Low	Intermediate
Index text	Were the index test results interpreted without knowledge of the results of the reference standard?	No	No	No	Yes	No	No	Yes	No	Yes	No	No
	If a threshold was used, was it pre-specified?	Yes	No	No	Yes	No	No	Yes	Yes	Yes	Yes	No
	Could the conduct or interpretation of the index test have introduced bias?	Intermediate	Low	Low	High	Low	Low	High	Intermediate	High	Intermediate	Low
Reference standard	Is the reference standard likely to correctly classify the target condition?	No	Intermediate	No	Intermediate	Intermediate	Intermediate	Intermediate	Intermediate	Intermediate	No	No
	Were the reference standard results interpreted without knowledge of the results of the index test?	No	No	No	Intermediate	Intermediate	Intermediate	Intermediate	Intermediate	No	No	No
	Could the reference standard, its conduct, or its interpretation have introduced bias?	High	High	High	Intermediate	Intermediate	Intermediate	Intermediate	Intermediate	High	High	High
Flow and timing	Was there an appropriate interval between index test(s) and reference standard?	Yes	Yes	Unclear	Yes	Yes	Yes	Yes	Yes	Yes	Yes	No
	Did all patients receive a reference standard?	Yes	Yes	Yes	Yes	No	No	Yes	Yes	Unclear	Yes	Yes
	Did all patients receive the same reference standard?	Yes	Yes	Yes	Yes	Yes	Yes	Yes	Yes	Unclear	Yes	Yes
	Were all patients included in the analysis?	Yes	Yes	Yes	No	No	Yes	Yes	Yes	Yes	Yes	Yes
	Could the patient flow have introduced bias?	Low	Low	Low	Intermediate	High	Intermediate	Low	Low	Intermediate	Low	Intermediate
**Overall**		**Low**	**High**	**High**	**Unclear**	**High**	**High**	**Unclear**	**Low**	**High**	**Unclear**	**Unclear**

Supplementary Table S4 Reasons for exclusion after full tests screening

Number	Reasons/study
**Reason 1**	**Modeling study(n=18)**
1	Cheng, P.; Wang, L.; Gong, W. In silico Analysis of Peptide-Based Biomarkers for the Diagnosis and Prevention of Latent Tuberculosis Infection. Front Microbiol 2022, 13, 947852, doi:10.3389/fmicb.2022.947852.
2	Davidow, A.L. Interferon-gamma release assay test characteristics depend upon the prevalence of active tuberculosis. Int J Tuberc Lung Dis 2009, 13, 1411-1415.
3	Fröberg, G.; Borgström, E.W.; Chryssanthou, E.; Correia-Neves, M.; Källenius, G.; Bruchfeld, J. A new mathematical model to identify contacts with recent and remote latent tuberculosis. ERJ Open Research 2019, 5, doi:10.1183/23120541.00078-2019.
4	Huang, H.L.; Lee, J.Y.; Lo, Y.S.; Liu, I.H.; Huang, S.H.; Huang, Y.W.; Lee, M.R.; Lee, C.H.; Cheng, M.H.; Lu, P.L.; et al. Whole-Blood 3-Gene Signature as a Decision Aid for Rifapentine-based Tuberculosis Preventive Therapy. Clinical Infectious Diseases 2022, 75, 743-752, doi:10.1093/cid/ciac003.
5	Kaul, S.; Nair, V.; Birla, S.; Dhawan, S.; Rathore, S.; Khanna, V.; Lohiya, S.; Ali, S.; Mannan, S.; Rade, K.; et al. Latent Tuberculosis Infection Diagnosis among Household Contacts in a High Tuberculosis-Burden Area: a Comparison between Transcript Signature and Interferon Gamma Release Assay. Microbiology Spectrum 2022, 10, doi:10.1128/spectrum.02445-21.
6	Lee, S.W.; Wu, L.S.; Huang, G.M.; Huang, K.Y.; Lee, T.Y.; Weng, J.T. Gene expression profiling identifies candidate biomarkers for active and latent tuberculosis. BMC Bioinformatics 2016, 17 Suppl 1, 3, doi:10.1186/s12859-015-0848-x.
7	Lin, Y.; Zhang, Y.; Yu, H.; Tian, R.; Wang, G.; Li, F. Identification of unique key genes and miRNAs in latent tuberculosis infection by network analysis. Mol Immunol 2019, 112, 103-114, doi:10.1016/j.molimm.2019.04.032.
8	Lloyd, T.; Steigler, P.; Mpande, C.A.M.; Rozot, V.; Mosito, B.; Schreuder, C.; Reid, T.D.; Hatherill, M.; Scriba, T.J.; Little, F.; et al. Multidimensional analysis of immune responses identified biomarkers of recent Mycobacterium tuberculosis infection. Plos Computational Biology 2021, 17, doi:10.1371/journal.pcbi.1009197.
9	Lv, L.; Li, C.; Zhang, X.; Ding, N.; Cao, T.; Jia, X.; Wang, J.; Pan, L.; Jia, H.; Li, Z.; et al. RNA Profiling Analysis of the Serum Exosomes Derived from Patients with Active and Latent Mycobacterium tuberculosis Infection. Front Microbiol 2017, 8, 1051, doi:10.3389/fmicb.2017.01051.

10 Ndzi, E.N.; Nkenfou, C.N.; Pefura, E.W.Y.; Mekue, L.C.M.; Guiedem, E.; Nguefeu, C.N.; Ngoufack, M.N.; Elong, E.; Yatchou, L.G.; Ndjolo, A.; et al. Tuberculosis diagnosis: algorithm that May discriminate latent from active tuberculosis. Heliyon 2019, 5, e02559, doi:10.1016/j.heliyon.2019.e02559.

11 Penn-Nicholson, A.; Hraha, T.; Thompson, E.G.; Sterling, D.; Mbandi, S.K.; Wall, K.M.; Fisher, M.; Suliman, S.; Shankar, S.; Hanekom, W.A.; et al. Discovery and validation of a prognostic proteomic signature for tuberculosis progression: A prospective cohort study. PLoS Med 2019, 16, e1002781, doi:10.1371/journal.pmed.1002781.

12 Song, Q.; Bian, Q.; Liang, T.; Zhang, Y.; Zhang, K. Identification of immune-related genes and susceptible population of pulmonary tuberculosis by constructing TF-miRNA-mRNA regulatory network. 2021, 131, doi:10.1016/j.tube.2021.102139.

13 Suliman, S.; Thompson, E.G.; Sutherland, J.; Weiner, J., 3rd; Ota, M.O.C.; Shankar, S.; Penn-Nicholson, A.; Thiel, B.; Erasmus, M.; Maertzdorf, J.; et al. Four-Gene Pan-African Blood Signature Predicts Progression to Tuberculosis. Am J Respir Crit Care Med 2018, 197, 1198-1208, doi:10.1164/rccm.201711-2340OC.

14 Sumner, T.; Mendelsohn, S.C.; Scriba, T.J.; Hatherill, M.; White, R.G. The impact of blood transcriptomic biomarker targeted tuberculosis preventive therapy in people living with HIV: a mathematical modelling study. BMC Medicine 2021, 19, doi:10.1186/s12916-021-02127-w.

15 Sun, H.; Pan, L.; Jia, H.; Zhang, Z.; Gao, M.; Huang, M.; Wang, J.; Sun, Q.; Wei, R.; Du, B.; et al. Label-Free Quantitative Proteomics Identifies Novel Plasma Biomarkers for Distinguishing Pulmonary Tuberculosis and Latent Infection. Front Microbiol 2018, 9, 1267, doi:10.3389/fmicb.2018.01267.

16 Weiner, J.; Domaszewska, T.; Donkor, S.; Kaufmann, S.H.E.; Hill, P.C.; Sutherland, J.S. Changes in transcript, metabolite, and antibody reactivity during the early protective immune response in humans to mycobacterium tuberculosis infection. Clinical Infectious Diseases 2020, 71, 30-40, doi:10.1093/cid/ciz785.

17 Weiner, J., 3rd; Maertzdorf, J.; Sutherland, J.S.; Duffy, F.J.; Thompson, E.; Suliman, S.; McEwen, G.; Thiel, B.; Parida, S.K.; Zyla, J.; et al. Metabolite changes in blood predict the onset of tuberculosis. Nat Commun 2018, 9, 5208, doi:10.1038/s41467-018-07635-7.

18 Yan, H.; Liu, G.; Liang, Y.; Wu, W.; Xia, R.; Jiao, L.; Shen, H.; Jia, Z.; Wang, Q.; Wang, Z.; et al. Up-regulated long noncoding RNA AC007128.1 and its genetic polymorphisms associated with Tuberculosis susceptibility. Annals of Translational Medicine 2021, 9, doi:10.21037/atm-21-2724.

Reason 2 No biomarkers were testing(n=5)

1 Kim, H.; Kim, S.H.; Jung, J.H.; Kim, M.J.; Kim, H.; Shin, S.; Chong, Y.P.; Kim, Y.H.; Lee, S.O.; Choi, S.H.; et al. The usefulness of quantitative interferon-gamma releasing assay response for predicting active tuberculosis in kidney transplant recipients: A quasi-experimental study. Journal of Infection 2020, 81, 403-410, doi:10.1016/j.jinf.2020.06.070.

2 Kim, H.C.; Jo, K.W.; Jung, Y.J.; Yoo, B.; Lee, C.K.; Kim, Y.G.; Yang, S.K.; Byeon, J.S.; Kim, K.J.; Ye, B.D.; et al. Diagnosis of latent tuberculosis infection before initiation of anti-tumor necrosis factor therapy using both tuberculin skin test and QuantiFERON-TB Gold In Tube assay. Scandinavian Journal of Infectious Diseases 2014, 46, 763-769, doi:10.3109/00365548.2014.938691.

3 Laundy, N.; Colley, S.; Fawcett, J.; Ryder, L.; Vedio, A.; Cohen, D.; Collini, P. Assessment of latent tuberculosis infection pre-immunomodulatory therapy; 5 year experience in a UK centre. Clinical Infection in Practice 2022, 13, doi:10.1016/j.clinpr.2022.100136.

4 Slouma, M.; Mahmoud, I.; Saidane, O.; Bouden, S.; Abdelmoula, L. Latent tuberculosis infection screening prior to biological treatment in Tunisian patients. Therapie 2017, 72, 573-578, doi:10.1016/j.therap.2017.02.002.

5 van Halsema, C.L.; Fielding, K.L.; Chihota, V.N.; Russell, E.C.; Lewis, J.J.C.; Churchyard, G.J.; Grant, A.D. Tuberculosis outcomes and drug susceptibility in individuals exposed to isoniazid preventive therapy in a high HIV prevalence setting. Aids 2010, 24, 1051-1055, doi:10.1097/QAD.0b013e32833849df.

Reason 3 Without serial data on treatment monitoring (n=9)

1 Araujo, L.S.; Mello, F.C.; Silva Nde, B.; Leung, J.A.; Machado, S.M.; Sardella, I.G.; Maciel Rde, M.; Saad, M.H. Evaluation of gamma interferon immune response elicited by the newly constructed PstS-1(285-374):CFP10 fusion protein to detect Mycobacterium tuberculosis infection. Clin Vaccine Immunol 2014, 21, 552-560, doi:10.1128/cvi.00726-13.

2 Scriba, T.J.; Fiore-Gartland, A.; Penn-Nicholson, A.; Mulenga, H.; Mbandi, S.K.; Borate, B.; Mendelsohn, S.C.; Hadley, K.; Hikuam, C.; Kaskar, M.; et al. Biomarker-guided tuberculosis preventive therapy (CORTIS): a randomised controlled trial. Lancet Infectious Diseases 2021, 21, 354-365, doi:10.1016/s1473-3099(20)30914-2.

3 Bakir, M.; Millington, K.A.; Soysal, A.; Deeks, J.J.; Efee, S.; Aslan, Y.; Dosanjh, D.P.; Lalvani, A. Prognostic value of a T-cell-based, interferon-gamma biomarker in children with tuberculosis contact. Ann Intern Med 2008, 149, 777-787, doi:10.7326/0003-4819-149-11-200812020-00248.

4 Chaisson, L.H.; Saraceni, V.; Cohn, S.; Seabrook, D.; Cavalcante, S.C.; Chaisson, R.E.; Golub, J.E.; Durovni, B. CD4(+)cell count stratification to guide tuberculosis preventive therapy for people living with HIV. Aids 2020, 34, 139-147, doi:10.1097/qad.0000000000002398.

5 Delemarre, E.M.; van Hoorn, L.; Bossink, A.W.J.; Drylewicz, J.; Joosten, S.A.; Ottenhoff, T.H.M.; Akkerman, O.W.; Goletti, D.; Petruccioli, E.; Navarra, A.; et al. Serum Biomarker Profile Including CCL1, CXCL10, VEGF, and Adenosine Deaminase Activity Distinguishes Active From Remotely Acquired Latent Tuberculosis. Frontiers in Immunology 2021, 12, doi:10.3389/fimmu.2021.725447.

6 Johnson, J.L.; Geldenhuys, H.; Thiel, B.A.; Toefy, A.; Suliman, S.; Pienaar, B.; Chheng, P.; Scriba, T.; Boom, W.H.; Hanekom, W.; et al. Effect of isoniazid therapy for latent TB infection on QuantiFERON-TB gold in-tube responses in adults with positive tuberculin skin test results in a high TB incidence area: a controlled study. Chest 2014, 145, 612-617, doi:10.1378/chest.13-1232.

7 Jung, Y.J.; Lee, J.Y.; Jo, K.W.; Yoo, B.; Lee, C.K.; Kim, Y.G.; Yang, S.K.; Byeon, J.S.; Kim, K.J.; Ye, B.D.; et al. The @either test positive@strategy for latent tuberculous infection before anti-tumour necrosis factor treatment. International Journal of and Lung Disease 2014, 18, 428-434, doi:10.5588/ijtld.13.0644.

8 Lee, H.; Park, H.Y.; Jeon, K.; Jeong, B.H.; Hwang, J.W.; Lee, J.; Cha, H.S.; Koh, E.M.; Kang, E.S.; Koh, W.J. QuantiFERON-TB gold in-tube assay for screening arthritis patients for latent tuberculosis infection before starting anti-tumor necrosis factor treatment. PLoS ONE 2015, 10, doi:10.1371/journal.pone.0119260.

9 Munoz, L.; Casas, S.; Juanola, X.; Bordas, X.; Martinez, C.; Santin, M.; Bellvitge Univ, H. Prevention of Anti-Tumor Necrosis Factor-Associated Tuberculosis: A 10-Year Longitudinal Cohort Study. Clinical Infectious Diseases 2015, 60, 349-356, doi:10.1093/cid/ciu796.

Reason 4 **No information on preventive treatment(n=59)**

1 Anterasian, C.; Warr, A.J.; Lacourse, S.M.; Kinuthia, J.; Richardson, B.A.; Nguyen, F.K.; Matemo, D.; Maleche-Obimbo, E.; Stewart, G.C.J.; Hawn, T.R. Non-IFN gamma Whole Blood Cytokine Responses to Mycobacterium tuberculosis Antigens in HIV-exposed Infants. Pediatric Infectious Disease Journal 2021, 40, 922-929, doi:10.1097/inf.0000000000003254.

2 Araujo, L.S.; da Silva, N.B.M.; da Silva, R.J.; Leung, J.A.M.; Mello, F.C.Q.; Saad, M.H.F. Profile of interferon-gamma response to latency-associated and novel in vivo expressed antigens in a cohort of subjects recently exposed to Mycobacterium tuberculosis. Tuberculosis (Edinb) 2015, 95, 751-757, doi:10.1016/j.tube.2015.08.002.

3 Baricevic, D.; Grle, S.P.; Vergles, J.M.; Cavka, S.C.; Jakopovic, M.; Redzepi, G.; Boras, Z.; Baricevic, M.; Samarzija, M. QuantiFERON-TB Gold In-Tube TEST IN THE DIAGNOSIS OF LATENT TUBERCULOSIS INFECTION IN ARTHRITIS PATIENTS TREATED WITH TUMOR NECROSIS FACTOR ANTAGONISTS. Acta Clinica Croatica 2017, 56, 203-209, doi:10.20471/acc.2017.56.02.02.

4 Cao, S.H.; Chen, Y.Q.; Sun, Y.; Liu, Y.; Zheng, S.H.; Zhang, Z.G.; Li, C.Y. Screening of Serum Biomarkers for Distinguishing between Latent and Active Tuberculosis Using Proteome Microarray. Biomed Environ Sci 2018, 31, 515-526, doi:10.3967/bes2018.069.

5 Chang, S.Y.; Chen, M.L.; Lee, M.R.; Liang, Y.C.; Lu, T.P.; Wang, J.Y.; Yan, B.S. SP110 Polymorphisms Are Genetic Markers for Vulnerability to Latent and Active Tuberculosis Infection in Taiwan. Dis Markers 2018, 2018, 4687380, doi:10.1155/2018/4687380.

6 Chapman, A.L.; Munkanta, M.; Wilkinson, K.A.; Pathan, A.A.; Ewer, K.; Ayles, H.; Reece, W.H.; Mwinga, A.; Godfrey-Faussett, P.; Lalvani, A. Rapid detection of active and latent tuberculosis infection in HIV-positive individuals by enumeration of Mycobacterium tuberculosis-specific T cells. Aids 2002, 16, 2285-2293, doi:10.1097/00002030-200211220-00008.

7 Clifford, V.; Tebruegge, M.; Zufferey, C.; Germano, S.; Forbes, B.; Cosentino, L.; Matchett, E.; McBryde, E.; Eisen, D.; Robins-Browne, R.; et al. Cytokine biomarkers for the diagnosis of tuberculosis infection and disease in adults in a low prevalence setting. 2019, 114, 91-102, doi:10.1016/j.tube.2018.08.011.

8 Connell, T.G.; Curtis, N.; Ranganathan, S.C.; Buttery, J.P. Performance of a whole blood interferon gamma assay for detecting latent infection with Mycobacterium tuberculosis in children. Thorax 2006, 61, 616-620, doi:10.1136/thx.2005.048033.

9 De Groote, M.A.; Higgins, M.; Hraha, T.; Wall, K.; Wilson, M.L.; Sterling, D.G.; Janjic, N.; Reves, R.; Ochsner, U.A.; Belknap, R. Highly Multiplexed Proteomic Analysis of Quantiferon Supernatants To Identify Biomarkers of Latent Tuberculosis Infection. J Clin Microbiol 2017, 55, 391-402, doi:10.1128/jcm.01646-16.

10 del Corral, H.; París, S.C.; Marín, N.D.; Marín, D.M.; López, L.; Henao, H.M.; Martínez, T.; Villa, L.; Barrera, L.F.; Ortiz, B.L.; et al. IFNgamma response to Mycobacterium tuberculosis, risk of infection and disease in household contacts of tuberculosis patients in Colombia. PloS one 2009, 4, e8257.

11 Demissie, A.; Leyten, E.M.S.; Abebe, M.; Wassie, L.; Aseffa, A.; Abate, G.; Fletcher, H.; Owiafe, P.; Hill, P.C.; Brookes, R.; et al. Recognition of stage-specific mycobacterial antigens differentiates between acute and latent infections with Mycobacterium tuberculosis. Clinical and Vaccine Immunology 2006, 13, 179-186, doi:10.1128/CVI.13.2.179-186.2006.

12 Deng, J.; Liu, L.; Yang, Q.; Wei, C.; Zhang, H.; Xin, H.; Pan, S.; Liu, Z.; Wang, D.; Liu, B.; et al. Urinary metabolomic analysis to identify potential markers for the diagnosis of tuberculosis and latent tuberculosis. Arch Biochem Biophys 2021, 704, 108876, doi:10.1016/j.abb.2021.108876.

13 Dhanasekaran, S.; Jenum, S.; Stavrum, R.; Ritz, C.; Faurholt-Jepsen, D.; Kenneth, J.; Vaz, M.; Grewal, H.M.; Doherty, T.M. Identification of biomarkers for Mycobacterium tuberculosis infection and disease in BCG-vaccinated young children in Southern India. Genes Immun 2013, 14, 356-364, doi:10.1038/gene.2013.26.

14 Dhanasekaran, S.; Jenum, S.; Stavrum, R.; Ritz, C.; Kenneth, J.; Vaz, M.; Doherty, T.M.; Grewal, H.M. Concordant or discordant results by the tuberculin skin test and the quantiFERON-TB test in children reflect immune biomarker profiles. Genes and immunity 2014, 15, 265-274, doi:10.1038/gene.2014.13.

15 Dirix, V.; Dauby, N.; Hites, M.; Watelet, E.; Van Praet, A.; Godefroid, A.; Petit, E.; Singh, M.; Locht, C.; Mascart, F.; et al. Optimal Detection of Latent Mycobacterium tuberculosis Infection by Combined Heparin-Binding Hemagglutinin (HBHA) and Early Secreted Antigenic Target 6 (ESAT-6) Whole-Blood Interferon Gamma Release Assays. Journal of Clinical Microbiology 2022, 60, doi:10.1128/jcm.02443-21.

16 Druszczynska, M.; Seweryn, M.; Wawrocki, S.; Kowalewska-Pietrzak, M.; Pankowska, A.; Rudnicka, W. Cytokine biosignature of active and latent mycobacterium tuberculosis infection in children. Pathogens 2021, 10, doi:10.3390/pathogens10050517.

17 Du, Y.; Gao, X.; Yan, J.X.; Zhang, H.R.; Cao, X.F.; Feng, B.X.; He, Y.J.; Guo, T.L.; Xin, H.N.; Gao, L. Relationship between DNA Methylation Profiles and Active Tuberculosis Development from Latent Infection: a Pilot Study in Nested Case-Control Design. Microbiology Spectrum 2022, 10, doi:10.1128/spectrum.00586-22.

18 Essone, P.N.; Leboueny, M.; Siawaya, A.C.M.; Alame-Emane, A.K.; Biyogo, O.C.A.; Tadatsin, P.H.D.; Nzoghe, A.M.; Essamazokou, D.U.; Ndjindji, O.M.; Padzys, G.S.; et al. M. tuberculosis infection and antigen specific cytokine response in healthcare workers frequently exposed to tuberculosis. Scientific Reports 2019, 9, doi:10.1038/s41598-019-44294-0.

19 Gatechompol, S.; Sophonphan, J.; Kerr, S.J.; Ubolyam, S.; Avihingsanon, A.; van Leth, F.; Cobelens, F. Monocyte-to-lymphocyte ratio as a predictor of TB among people living with HIV. Int J Tuberc Lung Dis 2021, 25, 933-938, doi:10.5588/ijtld.21.0300.

20 Geng, X.; Wu, X.; Yang, Q.; Xin, H.; Zhang, B.; Wang, D.; Liu, L.; Liu, S.; Chen, Q.; Liu, Z.; et al. Whole transcriptome sequencing reveals neutrophils◎transcriptional landscape associated with active tuberculosis. Front Immunol 2022, 13, 954221, doi:10.3389/fimmu.2022.954221.

21 Ghanaie, R.M.; Karimi, A.; Azimi, L.; James, S.; Nasehi, M.; Mishkar, A.P.; Sheikhi, M.; Fallah, F.; Tabatabaei, S.R.; Hoseini-Alfatemi, S.M. Diagnosis of latent tuberculosis infection among pediatric household contacts of Iranian tuberculosis cases using tuberculin skin test, IFN- gamma release assay and IFN-gamma-induced protein-10. Bmc Pediatrics 2021, 21, doi:10.1186/s12887-021-02524-3.

22 Guggino, G.; Orlando, V.; Cutrera, S.; La Manna, M.P.; Di Liberto, D.; Vanini, V.; Petruccioli, E.; Dieli, F.; Goletti, D.; Caccamo, N. Granzyme A as a potential biomarker of Mycobacterium tuberculosis infection and disease. Immunol Lett 2015, 166, 87-91, doi:10.1016/j.imlet.2015.05.019.

23 Halliday, A.; Masonou, T.; Tolosa-Wright, M.R.; Guo, Y.; Hoang, L.; Parker, R.; Boakye, A.; Takwoingi, Y.; Badhan, A.; Jain, P.; et al. Defining the Role of Cellular Immune Signatures in

Diagnostic Evaluation of Suspected Tuberculosis. Journal of Infectious Diseases 2022, 225, 1632-1641, doi:10.1093/infdis/jiab311.

24 Hill, P.C.; Jackson-Sillah, D.J.; Fox, A.; Brookes, R.H.; de Jong, B.C.; Lugos, M.D.; Adetifa, I.M.; Donkor, S.A.; Aiken, A.M.; Howie, S.R.; et al. Incidence of Tuberculosis and the Predictive Value of ELISPOT and Mantoux Tests in Gambian Case Contacts. Plos One 2008, 3, doi:10.1371/journal.pone.0001379.

25 Hong, J.Y.; Kim, A.; Park, S.Y.; Cho, S.N.; Dockrell, H.M.; Hur, Y.G. Screening for Mycobacterium tuberculosis Infection Using Beijing/K Strain-Specific Peptides in a School Outbreak Cohort. Frontiers in Cellular and Infection Microbiology 2021, 11, doi:10.3389/fcimb.2021.599386.

26 Kim, A.; Park, K.J.; Kim, Y.S.; Cho, S.N.; Dockrell, H.M.; Hur, Y.G. Diagnostic potential of a ppe protein derived from mycobacterium tuberculosis beijing/k strain. Yonsei Medical Journal 2020, 61, 789-796, doi:10.3349/ymj.2020.61.9.789.

27 Lu, L.L.; Smith, M.T.; Yu, K.K.Q.; Luedemann, C.; Suscovich, T.J.; Grace, P.S.; Cain, A.; Yu, W.H.; McKitrick, T.R.; Lauffenburger, D.; et al. IFN-γ-independent immune markers of Mycobacterium tuberculosis exposure. Nature Medicine 2019, 25, 977-987, doi:10.1038/s41591-019-0441-3.

28 Machado, F.V.; Morais, C.; Santos, S.; Reis, R. Evaluation of CD8(+) response in QuantiFERON-TB Gold Plus as a marker of recent infection. Respiratory Medicine 2021, 185, doi:10.1016/j.rmed.2021.106508.

29 Moon, S.M.; Park, I.A.; Kim, S.M.; Park, S.J.; Jung, J.H.; Kim, Y.H.; Park, J.B.; Hong, B.; Lee, S.O.; Choi, S.H.; et al. Living donor and recipient screening for latent tuberculosis infection by tuberculin skin test and interferon-gamma releasing assay in a country with an intermediate burden of tuberculosis. Journal of Infection and Chemotherapy 2013, 19, 1009-1013, doi:10.1007/s10156-013-0578-9.

30 Mpande, C.A.M.; Musvosvi, M.; Rozot, V.; Mosito, B.; Reid, T.D.; Schreuder, C.; Lloyd, T.; Bilek, N.; Huang, H.; Obermoser, G.; et al. Antigen-Specific T-Cell Activation Distinguishes between Recent and Remote Tuberculosis Infection. American Journal of Respiratory and Critical Care Medicine 2021, 203, 1556-1565, doi:10.1164/rccm.202007-2686OC.

31 Ndiaye, B.P.; Thienemann, F.; Ota, M.; Landry, B.S.; Camara, M.; Dieye, S.; Dieye, T.N.; Esmail, H.; Goliath, R.; Huygen, K.; et al. Safety, immunogenicity, and efficacy of the candidate tuberculosis vaccine MVA85A in healthy adults infected with HIV-1: a randomised, placebo-controlled, phase 2 trial. Lancet Respiratory Medicine 2015, 3, 190-200, doi:10.1016/s2213-2600(15)00037-5.

32 O'Neal, S.; Hedberg, K.; Markum, A.; Schafer, S. Discordant tuberculin skin and interferon-gamma tests during contact investigations: a dilemma for tuberculosis controllers. Int J Tuberc Lung Dis 2009, 13, 662-664.

33 Ordway, D.J.; Pinto, L.; Costa, L.; Martins, M.; Leandro, C.; Viveiros, M.; Amaral, L.; Arroz, M.J.; Ventura, F.A.; Dockrell, H.M. Gamma delta T cell responses associated with the development of tuberculosis in health care workers. FEMS Immunol Med Microbiol 2005, 43, 339-350, doi:10.1016/j.femsim.2004.09.005.

34 Papay, P.; Eser, A.; Winkler, S.; Frantal, S.; Primas, C.; Miehsler, W.; Novacek, G.; Vogelsang, H.; Dejaco, C.; Reinisch, W. Factors impacting the results of interferon-γ release assay and tuberculin skin test in routine screening for latent tuberculosis in patients with inflammatory bowel diseases. Inflammatory Bowel Diseases 2011, 17, 84-90, doi:10.1002/ibd.21427.

35 Passalent, L.; Khan, K.; Richardson, R.; Wang, J.; Dedier, H.; Gardam, M. Detecting latent tuberculosis infection in hemodialysis patients: a head-to-head comparison of the T-SPOT.TB test, tuberculin skin test, and an expert physician panel. Clin J Am Soc Nephrol 2007, 2, 68-73, doi:10.2215/cjn.01280406.

36 Pérez-Recio, S.; Pallarès, N.; Grijota-Camino, M.D.; Sánchez-Montalvá, A.; Barcia, L.; Campos-Gutiérrez, S.; Pomar, V.; Rabuñal-Rey, R.; Balcells, M.E.; Gazel, D.; et al. Identification of Recent Tuberculosis Exposure Using QuantiFERON-TB Gold Plus, a Multicenter Study. Microbiol Spectr 2021, 9, e0097221, doi:10.1128/Spectrum.00972-21.

37 Raqib, R.; Kamal, S.M.M.; Rahman, M.J.; Rahim, Z.; Banu, S.; Bardhan, P.K.; Chowdhury, F.; Ara, G.; Zaman, K.; Breiman, R.F.; et al. Use of antibodies in lymphocyte secretions for detection of subclinical tuberculosis infection in asymptomatic contacts. Clinical and Diagnostic Laboratory Immunology 2004, 11, 1022-1027, doi:10.1128/CDLI.11.6.1022-1027.2004.

38 Reichler, M.R.; Hirsch, C.; Yuan, Y.; Khan, A.; Dorman, S.E.; Schluger, N.; Sterling, T.R. Predictive value of TNF-α, IFN-γ, and IL-10 for tuberculosis among recently exposed contacts in the United States and Canada. BMC Infect Dis 2020, 20, 553, doi:10.1186/s12879-020-05185-2.

39 Santin, M.; Morandeira-Rego, F.; Alcaide, F.; Rabuñal, R.; Anibarro, L.; Agüero-Balbín, R.; Casas-Garcia, X.; Pérez-Escolano, E.; Navarro, M.D.; Sánchez, F.; et al. Detection of interleukin-2 is not useful for distinguishing between latent and active tuberculosis in clinical practice: a prospective cohort study. Clin Microbiol Infect 2016, 22, 1007.e1001-1007.e1005, doi:10.1016/j.cmi.2016.09.004.

40 Sauzullo, I.; Scrivo, R.; Mengoni, F.; Ermocida, A.; Coppola, M.; Valesini, G.; Vullo, V.; Mastroianni, C.M. Multi-functional flow cytometry analysis of CD4+T cells as an immune biomarker for latent tuberculosis status in patients treated with tumour necrosis factor (TNF) antagonists. Clinical and Experimental Immunology 2014, 176, 410-417, doi:10.1111/cei.12290.

41 Shen, Y.; Ma, H.F.; Luo, D.; Guan, J.L. The T-SPOT.TB assay used for screening and monitoring of latent tuberculosis infection in patients with Behçet's disease pre- and post-anti-TNF treatment: A retrospective study. Journal of the Chinese Medical Association 2019, 82, 375-380, doi:10.1097/JCMA.0000000000000071.

42 Singer, S.N.; Ndumnego, O.C.; Kim, R.S.; Ndung@u, T.; Anastos, K.; French, A.; Churchyard, G.; Paramithiothis, E.; Kasprowicz, V.O.; Achkar, J.M. Plasma host protein biomarkers correlating with increasing Mycobacterium tuberculosis infection activity prior to tuberculosis diagnosis in people living with HIV. Ebiomedicine 2022, 75, doi:10.1016/j.ebiom.2021.103787.

43 Sivakumaran, D.; Jenum, S.; Ritz, C.; Vaz, M.; Doherty, T.M.; Grewal, H.M.S. Improving Assignments for Therapeutic and Prophylactic Treatment Within TB Households. A Potential for Immuno-Diagnosis? Frontiers in Immunology 2022, 13, doi:10.3389/fimmu.2022.801616.

44 Son, C.N.; Jun, J.B.; Kim, J.H.; Sung, I.H.; Yoo, D.H.; Kim, T.H. Follow-up testing of interferon-gamma release assays are useful in ankylosing spondylitis patients receiving anti-tumor necrosis factor alpha for latent tuberculosis infection. Journal of Korean medical science 2014, 29, 1090-1093, doi:10.3346/jkms.2014.29.8.1090.

45 Sutherland, J.S.; Jeffries, D.J.; Donkor, S.; Walther, B.; Hill, P.C.; Adetifa, I.M.; Adegbola, R.A.; Ota, M.O. High granulocyte/lymphocyte ratio and paucity of NKT cells defines TB disease in a TB-endemic setting. Tuberculosis (Edinb) 2009, 89, 398-404, doi:10.1016/j.tube.2009.07.004.

46 Talat, N.; Shahid, F.; Dawood, G.; Hussain, R. Dynamic changes in biomarker profiles associated with clinical and subclinical tuberculosis in a high transmission setting: A four-year follow-up study. Scandinavian Journal of Immunology 2009, 69, 537-546, doi:10.1111/j.1365-3083.2009.02250.x.

47 Teklu, T.; Kwon, K.; Wondale, B.; HaileMariam, M.; Zewude, A.; Medhin, G.; Legesse, M.; Pieper, R.; Ameni, G. Potential Immunological Biomarkers for Detection of Mycobacterium tuberculosis Infection in a Setting Where M. tuberculosis Is Endemic, Ethiopia. Infect Immun 2018, 86, doi:10.1128/iai.00759-17.

48 Topic, R.Z.; Dodig, S.; Zoricic-Letoja, I. Interferon-gamma and Immunoglobulins in Latent Tuberculosis Infection. Archives of Medical Research 2009, 40, 103-108, doi:10.1016/j.arcmed.2008.11.001.

49 Tsiouris, S.J.; Austin, J.; Toro, P.; Coetzee, D.; Weyer, K.; Stein, Z.; El-Sadr, W.M. Results of a tuberculosis-specific IFN-gamma assay in children at high risk for tuberculosis infection. Int J Tuberc Lung Dis 2006, 10, 939-941.

50 Tuuminen, T.; Salo, E.; Kotilainen, H.; Ruhwald, M. Evaluation of the filter paper IP-10 tests in school children after exposure to tuberculosis: a prospective cohort study with a 4-year follow-up. Bmj Open 2012, 2, doi:10.1136/bmjopen-2012-001751.

51 Wang, S.; Diao, N.; Lu, C.; Wu, J.; Gao, Y.; Chen, J.; Zhou, Z.; Huang, H.; Shao, L.; Jin, J.; et al. Evaluation of the Diagnostic Potential of IP-10 and IL-2 as Biomarkers for the Diagnosis of Active and Latent Tuberculosis in a BCG-Vaccinated Population. PLoS ONE 2012, 7, doi:10.1371/journal.pone.0051338.

52 Wu, L.; Hu, Y.; Li, D.; Jiang, W.; Xu, B. Screening toll-like receptor markers to predict latent tuberculosis infection and subsequent tuberculosis disease in a Chinese population. BMC Medical Genetics 2015, 16, doi:10.1186/s12881-015-0166-1.

53 Xin, H.N.; Cao, X.F.; Du, Y.; Yan, J.X.; He, R.; Liu, Z.S.; Zhang, H.R.; He, Y.; Zhang, B.; Wang, D.K.; et al. The Association between Circulating microRNAs and the Risk of Active Disease Development from Latent Tuberculosis Infection: a Nested Case-Control Study. Microbiology Spectrum 2022, 10, doi:10.1128/spectrum.02625-21.

54 Yan, Z.H.; Zheng, X.F.; Yi, L.; Wang, J.; Wang, X.J.; Wei, P.J.; Jia, H.Y.; Zhou, L.J.; Zhao, Y.L.; Zhang, H.T. CD137 is a Useful Marker for Identifying CD4(+) T Cell Responses to Mycobacterium tuberculosis. Scand J Immunol 2017, 85, 372-380, doi:10.1111/sji.12541.

55 Yang, Y.; Li, X.; Cui, W.; Guan, L.; Shen, F.; Xu, J.; Zhou, F.; Li, M.; Gao, C.; Jin, Q.; et al. Potential association of pulmonary tuberculosis with genetic polymorphisms of toll-like receptor 9 and interferon-gamma in a Chinese population. BMC Infectious Diseases 2013, 13, doi:10.1186/1471-2334-13-511.

56 Yassin, M.A.; Petrucci, R.; Garie, K.T.; Harper, G.; Arbide, I.; Aschalew, M.; Merid, Y.; Kebede, Z.; Bawazir, A.A.; Abuamer, N.M.; et al. Can interferon-gamma or interferon-gamma-induced-protein-10 differentiate tuberculosis infection and disease in children of high endemic areas? PLoS One 2011, 6, e23733, doi:10.1371/journal.pone.0023733.

57 Yuan, K.; Wu, X.Q.; Zhang, Q.; Zhong, Z.M.; Chen, J.T. Enzyme-linked immunospot assay response to recombinant CFP-10/ESAT-6 fusion protein among patients with spinal tuberculosis: implications for diagnosis and monitoring of surgical therapy. International Journal of Infectious Diseases 2013, 17, E733-E738, doi:10.1016/j.ijid.2013.02.027.

58 Zak, D.E.; Penn-Nicholson, A.; Scriba, T.J.; Thompson, E.; Suliman, S.; Amon, L.M.; Mahomed, H.; Erasmus, M.; Whatney, W.; Hussey, G.D.; et al. A blood RNA signature for tuberculosis disease risk: a prospective cohort study. Lancet 2016, 387, 2312-2322, doi:10.1016/s0140-6736(15)01316-1.

59 Coppola, M.; Villar-Hernández, R.; van Meijgaarden, K.E.; Latorre, I.; Muriel Moreno, B.; Garcia-Garcia, E.; Franken, K.; Prat, C.; Stojanovic, Z.; De Souza Galvão, M.L.; et al. Cell-Mediated Immune Responses to in vivo-Expressed and Stage-Specific Mycobacterium tuberculosis Antigens in Latent and Active Tuberculosis Across Different Age Groups. Front Immunol 2020, 11, 103, doi:10.3389/fimmu.2020.00103.

Reason 5 **Sample size<10 (n=1)**

1 Shu, D.; Zhang, Z.; Zhou, E.Y.; Ma, X.; Zhao, Y. Is chemoprophylaxis necessary for all latent tuberculosis infection patients receiving IL-17 inhibitors? A cohort study. Dermatologic Therapy 2020, 33, doi:10.1111/dth.14512.

Reason 6 **Unavailable data (n=3)**

1 Mayito, J.; Meya, D.B.; Rhein, J.; Sekaggya-Wiltshire, C. Utility of the monocyte to lymphocyte ratio in diagnosing latent tuberculosis among HIV-infected individuals with a negative tuberculosis symptom screen. Plos One 2020, 15, doi:10.1371/journal.pone.0241786.

2 Rangaka, M.X.; Hamada, Y.; Duong, T.; Bern, H.; Calvert, J.; Francis, M.; Clarke, A.L.; Ghanouni, A.; Hack, V.; Owen-Powell, E.; et al. Randomised controlled trial to evaluate the effectiveness of using the RD-1-based C-Tb skin test as a replacement for blood-based interferon-gamma release assay for detection of, and initiation of preventive treatment for, tuberculosis infection: RID-TB:Dx study protocol. Bmj Open 2021, 11, doi:10.1136/bmjopen-2021-050595.

3 Sali, M.; Buonsenso, D.; D◎Alfonso, P.; De Maio, F.; Ceccarelli, M.; Battah, B.; Palucci, I.; Chiacchio, T.; Goletti, D.; Sanguinetti, M.; et al. Combined use of Quantiferon and HBHA-based IGRA supports tuberculosis diagnosis and therapy management in children. Journal of Infection 2018, 77, 526-533, doi:10.1016/j.jinf.2018.09.011.

Reason 7 **Without data on treatment monitoring (n=10)**

1 Broderick, C.; Cliff, J.M.; Lee, J.S.; Kaforou, M.; Moore, D.A.J. Host transcriptional response to TB preventive therapy differentiates two sub-groups of IGRA-positive individuals. Tuberculosis 2021, 127, doi:10.1016/j.tube.2020.102033.

2 Cantini, F.; Lubrano, E.; Marchesoni, A.; Mathieu, A.; Olivieri, I.; Salvarani, C.; Scarpa, R.; Spadaro, A. Latent tuberculosis infection detection and active tuberculosis prevention in patients receiving anti-TNF therapy: an Italian nationwide survey. International Journal of Rheumatic Diseases 2016, 19, 799-805, doi:10.1111/1756-185x.12708.

3 Corbiere, V.; Pottier, G.; Bonkain, F.; Schepers, K.; Verscheure, V.; Lecher, S.; Doherty, T.M.; Locht, C.; Mascart, F. Risk Stratification of Latent Tuberculosis Defined by Combined Interferon Gamma Release Assays. Plos One 2012, 7, doi:10.1371/journal.pone.0043285.

4 Gatechompol, S.; Harnpariphan, W.; Supanan, R.; Suwanpimolkul, G.; Sophonphan, J.; Ubolyam, S.; Kerr, S.J.; Avihingsanon, A.; Kawkitinarong, K. Prevalence of latent tuberculosis infection and feasibility of TB preventive therapy among Thai prisoners: a cross-sectional study. Bmc Public Health 2021, 21, doi:10.1186/s12889-021-11271-0.

5 Jeong, D.H.; Kang, J.; Jung, Y.J.; Yoo, B.; Lee, C.K.; Kim, Y.G.; Hong, S.; Shim, T.S.; Jo, K.W. Comparison of latent tuberculosis infection screening strategies before tumor necrosis factor inhibitor treatment in inflammatory arthritis: IGRA-alone versus combination of TST and IGRA. PLoS ONE 2018, 13, doi:10.1371/journal.pone.0198756.

6. Kwan, P.K.W.; Periaswamy, B.; De Sessions, P.F.; Lin, W.; Molton, J.S.; Naftalin, C.M.; Naim, A.N.M.; Hibberd, M.L.; Paton, N.I. A blood RNA transcript signature for TB exposure in household contacts. BMC Infectious Diseases 2020, 20, doi:10.1186/s12879-020-05116-1.
7. Streitz, M.; Fuhrmann, S.; Powell, F.; Quassem, A.; Nomura, L.; Maecker, H.; Martus, P.; Volk, H.D.; Kern, F. Tuberculin-specific T cells are reduced in active pulmonary tuberculosis compared to LTBI or status post BCG vaccination. Journal of Infectious Diseases 2011, 203, 378-382, doi:10.1093/infdis/jiq065.
8. Valinetz, E.D.; Matemo, D.; Gersh, J.K.; Joudeh, L.L.; Mendelsohn, S.C.; Scriba, T.J.; Hatherill, M.; Kinuthia, J.; Wald, A.; Cangelosi, G.A.; et al. Isoniazid preventive therapy and tuberculosis transcriptional signatures in people with HIV. Aids 2022, 36, 1363-1371, doi:10.1097/qad.0000000000003262.
9. Weinberg, A.; Aaron, L.; Montepiedra, G.; Sterling, T.R.; Browning, R.; Mmbaga, B.; Vhembo, T.; Naik, S.; Kabugho, E.; Masheto, G.; et al. Effects of Pregnancy and Isoniazid Preventive Therapy on Mycobacterium tuberculosis Interferon Gamma Response Assays in Women With HIV. Clinical Infectious Diseases 2021, 73, E3555-E3562, doi:10.1093/cid/ciaa1083.
10. Wergeland, I.; Assmus, J.; Dyrhol-Riise, A.M. T Regulatory Cells and Immune Activation in Mycobacterium tuberculosis Infection and the Effect of Preventive Therapy. Scandinavian Journal of Immunology 2011, 73, 234-242, doi:10.1111/j.1365-3083.2010.02496.x.

Supplementary Table S5 List of included studies

Number	First author (Year)	Title
1	Ying Du (2022)	Association Between Plasma Exosomes S100A9/C4BPA and Latent Tuberculosis Infection Treatment: Proteomic Analysis Based on a Randomized Controlled Study
2	Mulugeta Belay (2021)	Detection of Mycobacterium tuberculosis complex DNA in CD34-positive peripheral blood mononuclear cells of asymptomatic tuberculosis contacts: an observational study
3	Xuefang Cao (2021)	The Association Between Mycobacteria-Specific Antigen-Induced Cytokines and Host Response to Latent Tuberculosis Infection Treatment in a Chinese Population
4	Ock-Hwa Kim (2020)	Comparison of the change in QuantiFERON-TB Gold Plus and QuantiFERON-TB Gold In-Tube results after preventive therapy for latent tuberculosis infection
5	Henan Xin (2020)	Dynamic changes of interferon gamma release assay results with latent tuberculosis infection treatment
6	Haoran Zhang (2020)	Serum level of IL-1ra was associated with the treatment of latent tuberculosis infection in a Chinese population
7	Elisa Petruccioli (2018)	Effect of therapy on Quantiferon-Plus response in patients with active and latent tuberculosis infection
8	Irene Andia Biraro (2015)	Effect of isoniazid preventive therapy on immune responses to mycobacterium tuberculosis: an open label randomised, controlled, exploratory study
9	SW Lee (2012)	Serial interferon-gamma release assays after chemoprophylaxis in a tuberculosis outbreak cohort
10	Delia Goletti (2007)	Isoniazid prophylaxis differently modulates T-cell responses to RD1-epitopes in contacts recently exposed to Mycobacterium tuberculosis: a pilot study
11	Katie Ewer (2006)	Dynamic antigen-specific point-source exposure to T-cell responses after Mycobacterium tuberculosis

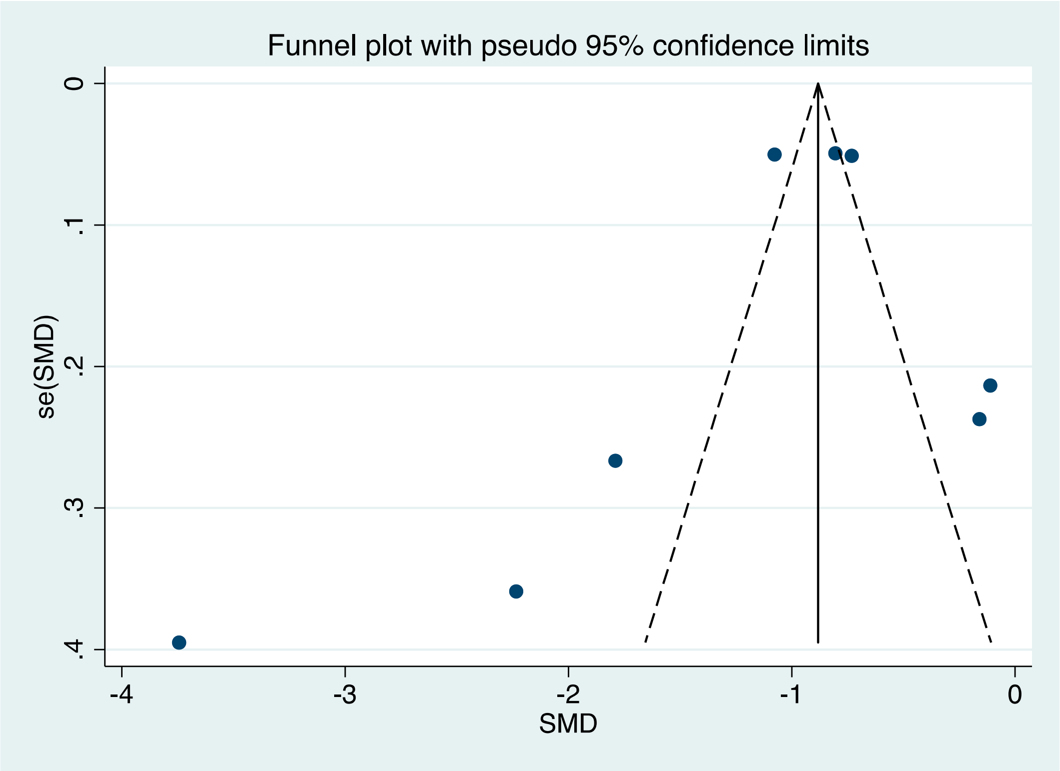

Supplementary Figure S1 Funnel plot for studies with INF-γ outcomes. The study specific SMD was plotted against their corresponding standard errors. Although Begg's rank correlation analysis (p=0.216) and Egger's weighted regression analysis (p=0.389) did not reach statistically significant, it was noticed that there was potential publication bias as asymmetry was present in the funnel plot.
